# Microglial replacement therapy: a potential therapeutic strategy for incurable *CSF1R*-related leukoencephalopathy

**DOI:** 10.1186/s40478-020-01093-3

**Published:** 2020-12-07

**Authors:** Jinming Han, Heela Sarlus, Zbigniew K. Wszolek, Virginija Danylaité Karrenbauer, Robert A. Harris

**Affiliations:** 1 Applied Immunology and Immunotherapy, Department of Clinical Neuroscience, Karolinska Institutet, Center for Molecular Medicine, Karolinska University Hospital, Solna, Sweden; 2 Department of Neurology, Mayo Clinic, Jacksonville, USA; 3 Department of Clinical Neuroscience, Karolinska Institutet, Stockholm, Sweden; 4 Department of Neurology, Karolinska University Hospital, Stockholm, Sweden

**Keywords:** *CSF1R*-related leukoencephalopathy, Microglia, Microglial replacement

## Abstract

*CSF1R*-related leukoencephalopathy is an adult-onset leukoencephalopathy with axonal spheroids and pigmented glia caused by colony stimulating factor 1 receptor (CSF1R) gene mutations. The disease has a global distribution and currently has no cure. Individuals with *CSF1R*-related leukoencephalopathy variably present clinical symptoms including cognitive impairment, progressive neuropsychiatric and motor symptoms. CSF1R is predominantly expressed on microglia within the central nervous system (CNS), and thus *CSF1R*-related leukoencephalopathy is now classified as a CNS primary microgliopathy. This urgent unmet medical need could potentially be addressed by using microglia-based immunotherapies. With the rapid recent progress in the experimental microglial research field, the replacement of an empty microglial niche following microglial depletion through either conditional genetic approaches or pharmacological therapies (CSF1R inhibitors) is being studied. Furthermore, hematopoietic stem cell transplantation offers an emerging means of exchanging dysfunctional microglia with the aim of reducing brain lesions, relieving clinical symptoms and prolonging the life of patients with *CSF1R*-related leukoencephalopathy. This review article introduces recent advances in microglial biology and *CSF1R*-related leukoencephalopathy. Potential therapeutic strategies by replacing microglia in order to improve the quality of life of *CSF1R*-related leukoencephalopathy patients will be presented.

## Introduction

*CSF1R*-related leukoencephalopathy, a subgroup of adult-onset leukodystrophy, is a progressive neurodegenerative white matter disease caused by mutations in the *colony stimulating factor 1 receptor (CSF1R)* gene [1]. CSF1R is highly expressed on microglia within the central nervous system (CNS) and thus *CSF1R*-related leukoencephalopathy is considered to be a primary CNS microgliopathy. The prognosis is extremely poor [2]. The terms of pigmented orthochromatic leukodystrophy (POLD) and hereditary diffuse leukoencephalopathy with spheroids (HDLS) were previously used to describe the disease spectrum [3, 4]. Specifically, HDLS was first reported in a Swedish family as early as 1984 based on the pathological hallmarks, including widespread white matter degeneration with numerous neuroaxonal spheroids, and the accumulation of lipid-laden and pigmented macrophages [5]. However, the alanyl tRNA synthetase (*AARS*) gene mutation has recently been identified in the original Swedish HDLS family, who have also been tested for *CSF1R* gene mutation status with negative results [6]. However, all POLD families tested for *CSF1R* gene mutations have been positive for the mutation [7]. To avoid further nomenclature confusion and adopting current nomenclature trends, we will use the term of *CSF1R*-related leukoencephalopathy, rather than HDLS or POLD, in this review.

Clinically, patients with *CSF1R*-related leukoencephalopathy typically manifest progressive cognitive decline, behavioral or personality changes and motor signs reminiscent of atypical Parkinsonism. *CSF1R*-related leukoencephalopathy may be misdiagnosed as frontotemporal dementia or dementia with Lewy bodies, cerebral autosomal dominant arteriopathy with subcortical infarcts and leukoencephalopathy (CADASIL), or primary progressive multiple sclerosis (MS), due to the overlapping clinical features and magnetic resonance imaging (MRI) abnormalities [8–10]. The age of disease onset in patients with *CSF1R*-related leukoencephalopathy varies from 10 to 78 years old with an average age of 43 years [4, 11]. The average survival is reported from 5.3 to 6.8 years [8, 12] but with rather wide range from 2 to 34 years [13]. Hyperintense signals of subcortical white matter in *CSF1R*-related leukoencephalopathy can rarely be extended to the cervical and thoracic spinal cord areas [14]. Optic nerve damage and myelitis may also occur in patients with *CSF1R*-related leukoencephalopathy, which confounds the diagnosis [9, 15]. Detailed clinical and radiological examinations are therefore of diagnostic importance. Konno and colleagues recently proposed the diagnostic criteria for *CSF1R*-related leukoencephalopathy, providing ‘probable’ or ‘possible’ designations for patients without a genetic test, and yielding high sensitivity and specificity for diagnosis [3]. Both genetic and pathological examinations are required for a confirmed diagnosis. It is important to note that there is no currently available, internationally used standardized and validated *CSF1R*-related leukoencephalopathy functional score scale. It is thus challenging to compare the evaluation of disease phenotypes and to evaluate experimental treatment clinical or surrogate endpoints due to the multifaceted clinical phenotypes.

**Fig. 1:**
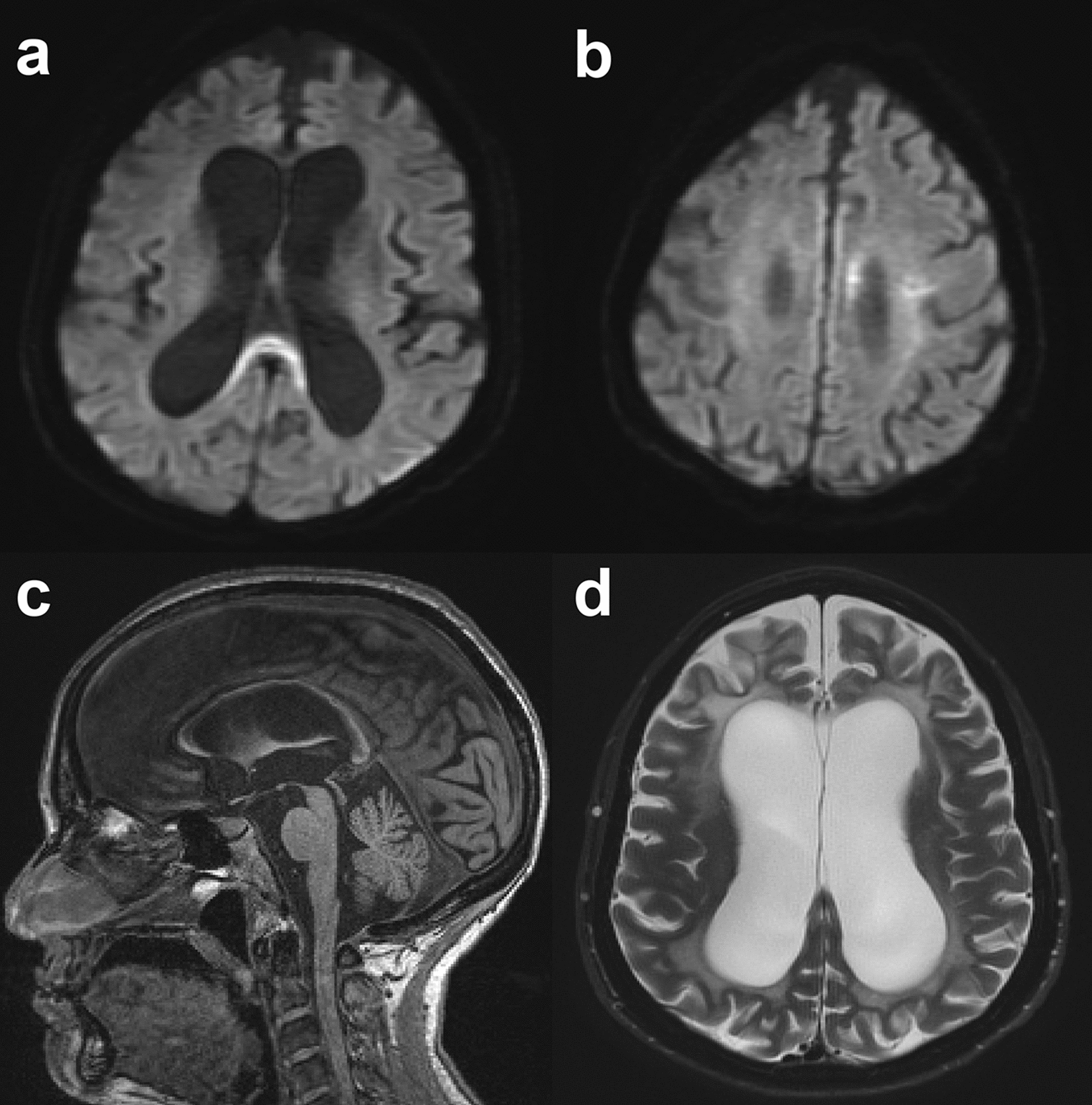
Brain MRI features of *CSF1R*-related leukoencephalopathy. The images were obtained from a 47-year-old female with progressive cognitive and behavioral deficits, and motor impairments with an approximate 3-year disease duration. Her father died of progressive dementia. The commercial genetic testing demonstrated the presence of heterozygote CSF1R mutation, p.L786S, c.2357 T > C. **a**, **b** Restricted diffusion in the splenium and left body of corpus callosum. **c** Thinning of corpus callosum on the sagittal section. **d** T2 hyperintensities in frontal and parietal white matter on the axial section. Courtesy of Daniel F. Broderick, M.D., Mayo Clinic Florida

A diagnostic value of unique calcifications distributed in selective brain regions on computed tomography scans has been proposed [16]. In addition, thinning of the corpus callosum, extensive non-confluent and occasionally even confluent subcortical white matter lesions on T2-weighted brain MRI images, and persistent restricted diffusion of diffuse-weighted imaging signal without gadolinium enhancement can be evident during the disease course. This may help in identifying potential *CSF1R*-related leukoencephalopathy cases (Fig. 1) [17]. We have previously demonstrated that the brain volume fractions measured by MRI did not differ significantly between *CSF1R*-related leukoencephalopathy and MS patients, while the cerebellum was relatively spared in *CSF1R*-related leukoencephalopathy patients [18]. We also employed an artificial intelligence machine learning to differentiate this disease state from MS using multi-parametric brain MRI images [19].

As there are no laboratory findings specific for *CSF1R*-related leukoencephalopathy, detailed clinical examinations are important to exclude differential diagnoses. Strikingly, neurofilament light chain, a marker of axonal injury [20], can serve as a potential biomarker for *CSF1R*-related leukoencephalopathy as higher levels are evident in both the serum and cerebrospinal fluid of these patients compared to in MS patients [21]. The level of neurofilament light chain already starts to increase in young *CSF1R* mutation carriers who exhibit no obvious clinical symptoms [21]. These features may assist in improving the diagnostic process and in differentiating *CSF1R*-related leukoencephalopathy white matter changes from other conditions. More importantly, it can serve as a potential biomarker for monitoring the response to therapeutic approaches.

The prevalence of *CSF1R*-related leukoencephalopathy is probably underestimated, but is gradually increasing mainly due to more widespread use of novel genetic tests such as next-generation sequencing [22]. As a genetic disease, *CSF1R*-related leukoencephalopathy causes emotional distress for patients and their families and poses a global burden for society. Patients can only be supported through maintaining nutrition and physical rehabilitation programs. Since microglia, the CNS-resident immune cells, are affected in this condition, developing immune cell-based therapies in order to improve disease prognosis and the quality of life of patients with *CSF1R*-related leukoencephalopathy represents a potential strategy to meet this urgent medical need.

### The role of CSF1R in *CSF1R*-related leukoencephalopathy and related mutant rodent models

The CSF1R cell-surface receptor is mainly expressed on microglia within the CNS [23], and as previously reviewed, the survival, maintenance and proliferation of microglia is critically dependent on CSF1R [24, 25]. Over 70 distinct mutations in the gene have been identified [2], most being located at the tyrosine kinase domain of *CSF1R* on chromosome 5 and encoded by exons 12–21 [4]. *CSF1R*-related leukoencephalopathy is supposed to be caused by the haploinsufficiency of *csf1r* gene [26]. Homozygous mutations of the *CSF1R* gene have recently been reported to cause a complete loss of microglia in the brain of an infant who died at 10 months of age [27].

*CSF1R* mutations cause a decreased activation of JNK, one of the downstream kinases involved in CSF1R signaling [28]. Expression of the CSF1R signaling ligands CSF-1 (predominant in the cerebellum) and interleukin (IL)-34 (predominant in the cortex) are important for microglial homeostasis in both mice and humans, indicating brain region-specific effects [29]. It is acknowledged that *CSF1R*-related leukoencephalopathy is caused by dysfunctional microglia, and that different mutation locations of the *CSF1R* gene lead to different clinical phenotypes of *CSF1R*-related leukoencephalopathy, with both disease presentation and progression varying significantly among family members [30, 31]. Although *CSF1R*-related leukoencephalopathy is a genetic disease inherited in an autosomal dominant pattern, approximately 36% of cases are sporadic [11]. This can be partly explained by the formation of mosaicism occurred at any cell division, allowing for survival and causing discrepancies of clinical phenotypes [32]. Other environmental factors may also contribute to sporadic cases.

As CSF1R is also expressed on other myeloid cells, *CSF1R* mutations may thus also affect myeloid cell function in the periphery and contribute to the pathogenesis [33–35]. Several novel observations support this idea. A defined *CSF1R* variant in one *CSF1R*-related leukoencephalopathy patient was associated with a progressive reduction in the frequency of circulating non-classical monocytes during disease course, since non-classical monocytes express the highest CSF1R levels [33]. Whether circulating non-classical monocytes infiltrate into the CNS or that they lose the ability to survive and proliferate in the circulation during disease progression remains unclear.

Microglia appear to be highly sensitive to the effects of *CSF1R* mutations [36]. While other tissue macrophages such as Langerhans cells in the skin and Kupffer cells in the liver also depend on CSF1R signaling [36], the effects of *CSF1R* mutations on these tissue macrophages in *CSF1R*-related leukoencephalopathy patients remains unknown. If additional factors or circulating monocytes may compensate for the loss of these tissue macrophages in *CSF1R*-related leukoencephalopathy requires further investigation.

Homozygous mutations of the *CSF1R* gene cause a generalized increase in bone density and metaphyseal dysplasia by influencing osteoclasts, suggesting that *CSF1R* deficiency may have an additional role in skeletal abnormalities [27]. CSF1R can also be expressed on some neurons and neuronal stem cells [2, 37]. Cux1 is a transcriptional factor associated with axonal projection and the numbers of Cux1^+^ neurons are reduced in *CSF1R*-related leukoencephalopathy patients compared to age-matched controls [27]. Potential effects of *CSF1R* mutations on these components, including impaired communication between microglia and neurons, warrant further investigation.

Rodent models of *CSF1R*-related leukoencephalopathy based on *Csf1r* mutations have been developed and exhibit some of the human disease features. The brain abnormalities of *Csf1r*^−/−^ mice are typically characterized by significant microglial loss and enlarged ventricles, but these mice die at a very young age. Conversely, *Csf1r*^+/−^ mice can survive during adulthood period but then develop abnormal symptoms including cognitive decline and depression with anxiety-like behavior [28]. Skeletal abnormalities are evident in *Csf1r*^−/−^ mice but not in *Csf1r*^+/−^ mice [28]. Unlike *Csf1r*^−/−^ mice, most *Csf1r*^−/−^ rats can survive into adulthood [38]. Despite Iba1^+^ microglia being absent in the brain of *Csf1r*^−/−^ rats there are few apparent structural brain abnormalities at 3 months of age. In addition, *Csf1r*^−/−^ rats exhibit postnatal growth retardation, skeletal abnormalities and infertility [38]. Furthermore, SIRPα^+^ circulating monocytes and CD68^+^ liver Kupffer cells are less affected in heterozygotic rats, but the numbers of these immune cells are significantly reduced in *Csf1r*^−/−^ homozygotic rats [38]. Overall, *Csf1r*-deficient rodent models on specific genetic backgrounds provide useful information for exploring the pathophysiology of *CSF1R*-related leukoencephalopathy and potential treatments.

### Pathological features of *CSF1R*-related leukoencephalopathy

Neuropathological features of *CSF1R*-related leukoencephalopathy include a widespread loss of myelin and axons, astrogliosis and macrophage accumulation in the presence of swollen and spherical axons [31]. Microglia are adapted to the CNS microenvironment and express specific gene signatures (*P2ry12*, *Tmem119* and *SiglecH*) in order to perform tissue-specific functions [39]. In *CSF1R*-related leukoencephalopathy, loss of Iba1^+^/P2ry12^+^ microglia were observed in vulnerable cerebral white matter, but not in unaffected grey matter [40]. Microglia exhibit a distinct morphology with narrow cytoplasm and fragmented processes containing beaded structures, differing from activated microglia that are characterized with retracted processes and enlarged cytoplasm in e.g. Alzheimer’s disease (AD) [41]. Microglial distribution has been suggested to be regulated by CSF1R [42], and the uneven distribution of microglia in the *CSF1R*-related leukoencephalopathy brain supports this notion [41]. While quantification of microglia in *CSF1R*-related leukoencephalopathy tissues is challenging, both immunohistochemical and western blot analyses indicate a reduction in microglial immunoreactivity in patient’s brains compared to in controls [41], despite a high proportion of microglia undergoing proliferation and particularly in areas with dense microglial occupation [41]. This suggests that microglia in *CSF1R*-related leukoencephalopathy retain the ability to proliferate, and that the reduction in their numbers might actually reflect reduced survival, given that CSF1R is important for microglial survival [43].

Infiltrating Iba1^+^P2ry12^−^ macrophages are detectable on histopathological examinations [4, 41], particularly in the degenerating corpus callosum, and most express CD68, CD163 and CD204 [41]. There is a dominance of CD163^+^ over CD68^+^ subpopulations present in the pigmented cells [31]. Interestingly, macrophages are evident in areas with sparsely distributed microglia, suggesting that macrophages are recruited to the degenerated white matter and might contribute to pathology. We have previously reported that long-term microglial depletion leads to monocyte colonization of the empty niche [44]. The functional role of infiltrating monocyte-derived cells in *CSF1R*-related leukoencephalopathy remains to be investigated [40].

### Sex differences in *CSF1R*-related leukoencephalopathy: do microglia play a role?

Although there is no sex difference in the prevalence of *CSF1R*-related leukoencephalopathy, it was reported that female patients develop clinical symptoms significantly earlier than do men [8, 12]. This interesting finding was further supported in a Chinese cohort containing 15 patients from 10 unrelated families, with younger age of disease onset in female than in male patients (34.2 vs. 39.2 years) [45]. Motor dysfunction was the most common initial symptom in women. Human sex hormones can mediate potential sex differences of microglia with exogenous estradiol enhancing humoral immunity while testosterone has an opposite effect [46]. The importance of sex-related differences in *CSF1R*-related leukoencephalopathy disease course requires further investigation [8].

Experimental studies have convincingly demonstrated sex-dependent structural and functional differences in microglia, which is drastically changing our viewpoint [47, 48]. Iba1^+^ microglial cell density in the cortex, hippocampus and amygdala is higher in adult male mice than in females [47]. Microglia in male mice express more MHC-related genes compared to females, suggesting that the antigen-presenting potential of microglia is distinct between males and females in mice [47]. Obvious transcriptional and translational properties of microglia were also noted between male and female mice [47].

Sex differences are more prominent in the settings of age-related disorders since microglial functionality becomes sexually divergent with aging [49]. Unlike in aged male mice, the expression of proinflammatory cytokines in the brain was increased in aged female mice in response to peripheral lipopolysaccharide (LPS) stimulation [50]. Brain-derived neurotrophic factor (BDNF) is produced by microglia and partial ablation of BDNF had different effects on proinflammatory cytokine production in the brains of male mice compared to females in response to LPS. This suggests that BDNF plays a role in mediating sex-dependent difference in intrathecal inflammatory responses to LPS [51].

Sex differences of microglial functions can be maintained even after they are transferred into the brain, as illustrated by transplantation of female microglia into the male brain, which conferred protection against stroke [48]. The impact of the microbiome on microglial properties is also sex-dependent, the long-term absence of the microbiome exhibiting more profound changes in females during adulthood [52]. Recently, we also observed sex-dependent severity of experimental autoimmune encephalomyelitis in adult mice following engraftment of microglia-like cells [53].

Although well documented, there are no consistent data proving sex-dependent effects during neurological diseases. However, these available preclinical results set the basis for further studies to explore how sex differences in human microglia might contribute to sex dependent phenotypic differences in *CSF1R*-related leukoencephalopathy patients. This may also provide translational evidence for developing potential microglial sex-specific therapies for *CSF1R*-related leukoencephalopathy.

### The microglial niche theory: a developing story

The macrophage niche theory, a novel concept first described in the macrophage research field, postulates that each macrophage cell occupies its own territory [54, 55] and that competitive repopulation of an empty macrophage niche is tightly sensed and regulated [44]. The depletion or death of tissue resident macrophages may provide an empty space, which triggers the proliferation of neighboring macrophages to repopulate the empty niche [55].

All tissue resident macrophage populations are established during development by embryonic precursors derived from either the yolk sac or from fetal liver monocytes [56]. In some tissues such as the brain, the embryonically derived macrophages maintain themselves during adulthood by local self-renewal [57], whereas in tissues such as the dermis, gut and pancreas the macrophages are maintained by continual replacement from the blood-circulating monocyte pool [58, 59]. Embryonic and adult macrophage precursors have similar abilities to differentiate into macrophages in diverse tissues, where each tissue accommodates a limited number of macrophage niches and the process of macrophage differentiation only halts once the niche is fully occupied [54].

Microglia in the CNS are unique in that the microglial precursors derived from immature erythromyeloid progenitors populate the CNS around embryonic day 8.0, before formation of the blood–brain barrier, and they differentiate locally into microglia [60]. Subsequent closure of the blood–brain barrier prevents further colonization of the CNS with additional pools of precursors. During homeostasis microglia remain separated from the circulation and maintain their numbers through local proliferation without replenishment by blood monocytes [57, 60]. Additional niches can be formed during organ growth during the neonatal period [54]. Microglial niches can become temporarily available under specific circumstances, such as following irradiation-mediated damage that can cause the leakiness of barriers and subsequent recruitment of immune cells from the peripheral pool [54, 61]. Furthermore, depletion using a selective inhibitor of CSF1R provides an almost empty microglia niche without disrupting the blood brain barrier [43]. The few remaining microglia repopulate the CNS through hyper-proliferation and exceed the original numbers of depleted microglia, which eventually normalizes to the baseline level [62, 63]. Depending on experimental depletion approaches, the newly repopulated microglia can either arise from remaining resident microglia [64] or infiltrating microglia-like cells [44, 65–67].

Kupffer cells enhance their proliferative ability to repopulate the niche when they are partly ablated [58]. A higher proliferative rate of microglia in selective brain regions has also been noted in *CSF1R*-related leukoencephalopathy patients through pathological examinations [41]. However, the numbers of microglia are significantly decreased in some but not all brain regions in patients, suggesting that signals involved in repopulating the microglial niche may be damaged in selective brain regions during *CSF1R*-related leukoencephalopathy [41]. Artificial administration of neurotrophic factors such as BDNF may also be necessary when the microglial niche is made available during *CSF1R*-related leukoencephalopathy [68, 69]. A better understanding of specific microglial niches and their associated regulatory molecular pathways is thus necessary in order to effectively design microglial replacement therapies for *CSF1R*-related leukoencephalopathy patients.

### Microglial replacement by the proliferation of resident microglia

The mechanisms of cell death including apoptosis, necroptosis and pyroptosis provide an avenue to stimulate the replacement of accumulated and unwanted cells with new cells in order to maintain proper tissue functions [70]. Specifically, microglia are self-maintained in the CNS through a balance between proliferation and apoptosis with little contribution from peripheral cells under physiological conditions [57]. Microglial depletion through conditional genetic depletion or pharmacological therapies is now considered as effective approach to permit new microglia to repopulate the CNS niche [25, 44]. By doing so, subsequent microglia replenishment might have the potential to compensate for the cell loss and resolve ongoing neuroinflammation [71].

Pharmacological targeting of microglia using CSF1R inhibitors such as PLX3397 [72], PLX5622 [73], BLZ945 [74], GW2850 [75], Ki20227 [76] and JNI-40346527 [77] are increasingly used to deplete microglia. These drugs can be integrated into rodent chow diets and eliminate microglia in the CNS by crossing the blood brain barrier without affecting the health and growth of adult mice [24, 78]. It was suggested that newly repopulating microglia following such depletion stemmed from the Nestin^+^ neuro-progenitors [43], while this was disputed in a later fate mapping study which indicated that repopulated microglia derive solely from the local proliferation of resident microglia [64]. Aged mice can restore their aged microglial cell densities and morphologies (exhibiting similar phenotypes as young microglia) following the proliferation of surviving microglia after PLX5622-mediated microglial depletion [71]. The newly repopulated microglia were beneficial in rescuing aged-related memory and long-term potentiation deficits in mice [71]. In support of this, a recent study demonstrated that repopulating microglia after experimental depletion using both genetic and pharmacological methods exhibit a pro-regenerative phenotype, which stimulated functional neurogenesis, improved spatial learning and memory deficits and promoted brain repair [79].

However, microglial depletion and repopulation by targeting either CSF1R or CX3CR1 may also affect CNS macrophages in the meninges or choroid, as well as other immune cells in the periphery [35, 80, 81]. In order to avoid potential effects on non-CNS resident macrophages, diphtheria toxin can be directly injected into the hippocampus to achieve selective microglia depletion in CX3CR1^CreERT2^ iDTR mice. In concordance with previous results, the appearance of repopulating microglia in the hippocampus contributes to improve the behavioral functions [79]. In order to avoid potential effects from the periphery, PLX3397 was administrated in primary organotypic hippocampal slice culture to specifically target microglia without involvement of peripheral myeloid cells [82]. Consistent with previous results, the newly repopulated microglia exhibited anti-inflammatory properties by up-regulating the expression of IL-10, CX3CL1 and BDNF [82]. Microglial repopulation can also down-regulate proinflammatory responses following LPS stimulation and persistent proinflammatory responses in the setting of chronic ethanol binging [82].

Different strategies for microglial replacement in the CNS or selective brain regions have been recently proposed [83]. Considering the beneficial effects of repopulating microglia in the CNS, it is tempting to hypothesize that replacing microglia in *CSF1R*-related leukoencephalopathy through a pharmacological depletion and repopulation paradigm may prolong the survival of patients, despite the newly repopulating microglia still carrying the *CSF1R* gene mutation. In preclinical studies, depletion of microglia can ameliorate cerebral white matter injury through reducing proinflammatory mediators [84]. Depleting microglia can also attenuate demyelination and neural damage in the mouse model carrying human *PLP1* mutations, resembling progressive MS [85].

We propose that a combination of microglial replacement by the proliferation of resident microglia after depletion and other anti-inflammatory treatments that modulate the microenvironment may be more effective for *CSF1R*-related leukoencephalopathy. As a novel and still developing field, potential safety concerns during microglial depletion and repopulation remain a challenge. In preclinical studies microglia depletion does not show obvious side-effects [78], while depleting prenatal microglia may lead to long-term behavioral abnormalities [86]. An open-label study in patients with amyotrophic lateral sclerosis in order to characterize the safety, tolerability and microglial response following pharmacological CSF1R inhibition with BLZ945 is still ongoing in Stockholm (ClinicalTrials.gov, identifier: NCT04066244). Although the primary endpoint was not reached in a phase II clinical trial, microglial depletion using PLX3397 with an oral dose 600 or 1000 mg/kg was well-tolerated in patients with recurrent glioblastoma, and was evident in tumor tissues [87, 88]. Some potential adverse events such as febrile neutropenia, drug-induced liver injury, anemia and hypertension were reported in some individuals [87]. It is also important to note that circulating CD14^dim^CD16^+^ monocytes (non-classical monocytes) are significantly reduced after PLX3397 treatment [87, 88]. Since the numbers of non-classical monocytes can also be decreased with disease progression in *CSF1R*-related leukoencephalopathy patients [33], the clinical translation of pharmacological CSF1R inhibitors should be considered with caution.

### Microglial replacement by infiltrating microglia-like cells

Apart from cell replacement by the proliferation of remaining host microglia following depletion [64], the engraftment of parenchymal microglia-like cells can be achieved under specific experimental circumstances once the microglial niche is made available [44, 65, 66]. We have previously demonstrated that microglia can be efficiently depleted (~ 95%) by the administration of tamoxifen in *CX3CR1*^*CreER/*+^*Rosa26*^*DTA/*+^ mice, causing intracellular DTA expression upon Cre recombination [44]. One month later the newly repopulated microglia in the CNS of these mice exhibited two distinct cell subpopulations differing in their expression of F4/80 [44]. Specifically, host microglia in the brain had a low expression of F4/80, while peripherally derived microglia-like cells in the brain expressed high levels of F4/80 and were P2ry12^−^ [44]. Along this line, a wave of Ly6C^hi^ monocytes were recruited into the brain of *Cx3cr1*^*CreER/*+^*Rosa26*^*DTA/*+^ mice only 2 days post- tamoxifen injection and before the appearance of F4/80^hi^ microglia-like cells in the brain [44]. Furthermore, intravenous adoptive transfer of cultured Ly6C^hi^ monocytes in microglia-depleted recipient mice led to the reconstitution of F4/80^hi^ microglia-like cells with little contribution from the F4/80^low^ (CNS-resident) microglial pool [44]. Our previous findings indicated that engrafted Ly6C^hi^ monocytes are shaped by the CNS microenvironment to gradually become microglial-like cells when the microglial niche is available after experimental ablation [54, 89], while remaining transcriptionally, epigenetically and functionally distinct from yolk-sac-derived resident microglia [44].

These findings are further supported by a recent study that convincingly demonstrated that graft-derived microglia-like cells can seed the brain parenchyma following irradiation, a condition that makes the microglial niche available [66]. Through local clonal expansion the monocyte-derived microglia-like cells competed with tissue resident microglia for space and expressed genes such as *Ccr2, Apoe*, *Ifnar1* and *Ms4a7 [*66]. As host-resident microglia and engrafted microglia-like cells yield distinct responses to LPS challenge [66], it is still important to question how a microglia-like cell repopulated CNS will function physiologically.

Basic research indicates that transplanted bone marrow-derived cells can rescue the phenotypes of *Csf1r*-null mice [89]. Bone marrow-derived microglia-like cells have a greater ability to clear cellular debris and dead cells compared to resident microglia, depending on different disease conditions [66]. Bone marrow-derived cells stimulated with CSF-1 upregulated microglial-specific surface markers such as Tmem119, and increased their functional ability to phagocytose amyloid-β peptides in vitro [90]. Hippocampal transplantation of bone marrow-derived microglia-like cells in an Alzheimer’s mouse model increased the phagocytic uptake of amyloid-β, leading to reduced amyloid-β burden in the brain and improved cognitive deficits [90]. Bone marrow-derived cells stimulated with granulocyte CSF (G-CSF) home and reside in the brain after radiation injury, modulating functional repair and restoring white matter damage [91].

**Fig. 2:**
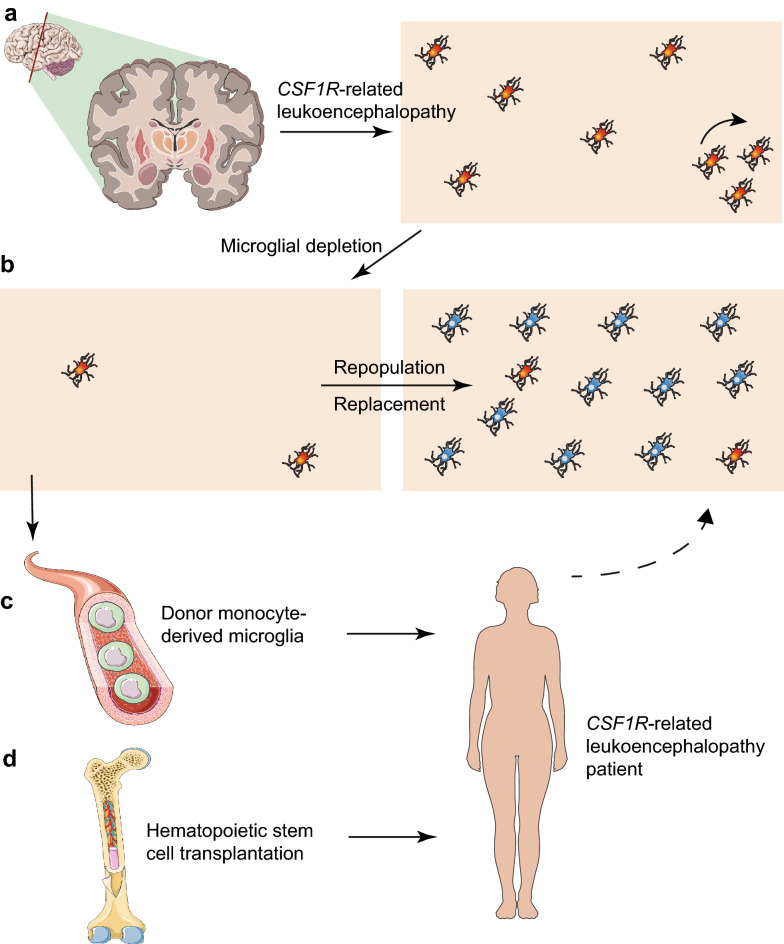
Potential scheme of microglial replacement therapy for *CSF1R*-related leukoencephalopathy. **a** Microglia lose the homeostatic phenotype and their numbers are decreased significantly in the brain tissues of patients with *CSF1R*-related leukoencephalopathy. Some microglia may undergo proliferation with a high proportion in selected brain regions. **b** The ablation of microglia using either pharmacological inhibition or genetic targeting makes the microglial niche available. Subsequently, the empty microglial niche can be repopulated (replaced) by donor monocytes-derived microglia-like cells (**c**) or hematopoietic stem cell transplantation (**d**)

Microglial replacement by infiltrating microglia-like cells thus provides an attractive possibility to remove dysfunctional microglia in patients with *CSF1R*-related leukoencephalopathy [25]. Furthermore, targeting the CX3CL1-CX3CR1 signaling pathway regulates the process of microglial repopulation from the proliferation of resident microglia in the CNS [92]. A combination of repopulation of microglia after depletion and bone marrow transplantation may serve as a novel therapeutic platform for *CSF1R*-related leukoencephalopathy. Clinically, the derivation of microglia-like cells from healthy donor circulating monocytes could be another option for replacing dysfunctional microglia (Fig. 2) [93]. However, the precise role of these engrafted microglia-like cells in the brain needs further investigation [94].

### Microglial replacement by hematopoietic stem cell transplantation

Empiric trials of glucocorticoids did not show obvious clinical improvements in reported *CSF1R*-related leukoencephalopathy cases [95]. Hematopoietic stem cell transplantation (HSCT) offers a potential regenerative strategy for patients in order to halt disease progression and improve survival (Fig. 2). A previous study reported an affected individual who underwent HSCT from her sibling remained stable for more than 15 years [96]. This meaningful finding is further supported by a recent study convincingly showing the effectiveness of HSCT from human leukocyte antigen-matched wild-type *CSF1R* gene donors in two *CSF1R*-related leukoencephalopathy patients [95]. These patients did not show any signs of acute or chronic graft-versus-host disease after HSCT, but experienced worsened neurological symptoms such as parkinsonism after transplantation [95]. During the follow-up period one patient developed pneumonia and the other suffered from new localization-related seizures. Reducing intensity-conditioning regimens is required in order to minimize potential toxicity during HSCT [95]. More importantly, the progression of cognitive decline, overall clinical outcomes and gait impairment gradually stabilized in 9 and 6 years post-HSCT for each patient [95]. Furthermore, in brain MRIs the T2 lesions and fluid-attenuated inversion recovery abnormalities were gradually reduced, and there was a significant resolution of abnormal multifocal reduced diffusion as measured by MRI after 2 years [95]. In both cases peripheral blood donor chimerism was successfully established after transplantation but the degree of chimerism in the CNS is not known since the patients are still alive [95]. Satisfactory long-term effects of HSCT on *CSF1R*-related leukoencephalopathy were noted in another case [97]. This patient also experienced deteriorated pyramidal symptoms 3 months after HSCT, while gradually improved during long-term follow-up [97].

Graft-derived ramified Iba1^+^ cells from a sex-mismatched donor was noted in post-mortem brains after HSCT through observation of Y-chromosomes using chromogenic in situ hybridization [66]. In an experimental model of demyelination, intraventricular transplantation of mesenchymal stem cells exerted neuroprotective effects by upregulating the expression of the CX3CL1/CX3CR1 axis, shifting proinflammatory microglia into an anti-inflammatory state and resulting in remyelination [98]. These labeled mesenchymal stem cells were present in the corpus callosum 2 weeks after transplantation [98]. In addition to their neurotrophic support and immunomodulation, stem cell grafts may also exercise beneficial effects through structural cell replacement [99]. The function of phagocytosis can also be modulated by grafted stem cells in animal models [99]. Transplantation of mesenchymal stem cells can promote disease recovery in an intraventricular hemorrhage rat model mainly by blocking reactive microglia, modulating the phosphorylation of STAT1 and p38 MAPK, and consequently reducing the production of proinflammatory cytokines [100].

All major brain cell types including microglia can be generated and differentiated from stem cells [101]. Although microglial replacement by HSCT is far from perfect, allogeneic HSCT could be beneficial in patients with *CSF1R*-related leukoencephalopathy in clinical practice by providing brain-engrafting microglia-like cells with donor wild-type CSF1R to repopulate the microglial niche [2]. However, to what extent HSCT can replace the resident microglia remains unclear.

Repopulating microglia from a human leukocyte antigen-matched donor could perform normal functions such as phagocytosis and cytokine production, while altered functions such as an increased release of cytokines after stimulation were noted in microglia-like cells derived from either a patient donor or a family member who also carried the *CSF1R* gene mutation [101]. Worsening clinical symptoms post-HSCT may be related to replacing microglia and phagocytosing debris in the brain. Finally, partial clinical stabilization and resolution of MRI abnormalities several years after HSCT may result from the replacement of some dysfunctional microglia by new microglia that are maintained in the CNS. It is also important to note that some early-onset *CSF1R*-related leukoencephalopathy patients did not show clinical symptom improvement following HSCT [102], suggesting that selection of the right patients, adequate design of clinical trials for rare diseases and long-term clinical monitoring with standardized, internationally validated tools will be necessary for evaluation of clinical outcomes and experimental treatment endpoints. Tissue resident macrophages in the CNS can be generated from stem cells, while only partially resembling embryonic-derived resident microglia [103]. Recreating the microglial niche and complex niche factors such as sex differences and cell-to cell communications should be considered when designing potential microglia replacement therapy.

## Concluding remarks

There are no available specific therapies for *CSF1R*-related leukoencephalopathy to date, and development of a potential microglia-based treatment is warranted. A detailed understanding of the microglial niche, and potential regulating factors such as epigenetic markers, novel pharmacological therapies to remove microglia by targeting CSF1R, emerging genomic engineering tools and stem cell treatments, may all facilitate the selective replacement of dysfunctional microglia with healthy microglia in *CSF1R*-related leukoencephalopathy patients. While still relatively novel, microglial replacement therapy is a scientifically sound strategy and presents an exciting research opportunity that could lead to clinical translation, alleviation of symptoms and improving the life quality of individuals who are suffering from currently incurable *CSF1R*-related leukoencephalopathy.

## Data Availability

Not applicable.

## Abbreviations

AARS: Alanyl tRNA synthetase
AD: Alzheimer’s disease
BDNF: Brain-derived neurotrophic factor
CADASIL: Cerebral autosomal dominant arteriopathy with subcortical infarcts and leukoencephalopathy
CNS: Central nervous system
CSF1R: Colony stimulating factor 1 receptor
HDLS: Hereditary diffuse leukoencephalopathy with spheroids
HSCT: Hematopoietic stem cell transplantation
G-CSF: Granulocyte CSF
LPS: Lipopolysaccharide
MRI: Magnetic resonance imaging
MS: Multiple sclerosis
POLD: Pigmented orthochromatic leukodystrophy
